# Phase Transitions and Switching Dynamics of Topological Domains in Hafnium Oxide-Based Cylindrical Ferroelectrics from Three-Dimensional Phase Field Simulation

**DOI:** 10.3390/nano15241901

**Published:** 2025-12-18

**Authors:** Pengying Chang, Hanxiao Zhang, Mengyao Xie, Huan Zhang, Yiyang Xie

**Affiliations:** Key Laboratory of Optoelectronics Technology of Ministry of Education, Beijing University of Technology, Beijing 100124, China

**Keywords:** ferroelectrics, cylindrical shell structure, phase field model, topological domain, phase transition, switching dynamics

## Abstract

The phase transitions and switching dynamics of topological polar textures in hafnium oxide (HfO_2_)-based cylindrical-shell ferroelectrics are studied using a three-dimensional (3D) phase field model based on the self-consistent solution of the time-dependent Ginzburg–Landau model and Poisson equation. The comprehensive interplays of bulk free energy, gradient energy, depolarization energy, and elastic energy are taken into account. When a cylindrical ferroelectric device is biased under the in-plane radial electric field, there is a size-controlled phase transition between the ferroelectric (FE), antiferroelectric (AFE), and paraelectric (PE) phases, depending on ferroelectric film thickness and cylindrical shell radius. For in-plane polarization textures at the equilibriums, the FE phase has a Néel-like texture with a center-type four-quad domain, the AFE phase has a monodomain texture, and the PE phase has a Bloch-like texture with a vortex four-quad domain. These polarization domain textures are resultant from energy competition and topologically protected by the geometrical confinement. The polarization dynamics from polar states towards equilibriums are analyzed considering the separated contributions of x- and y-components of polarizations that are driven by x-y in-plane electric fields. The emergent topological domains and phase transitions provide guidelines for geometrical engineering of a novel nano-structured ferroelectric device that is different from the planar one, offering new possibilities for multi-functional high-density ferroelectric memory.

## 1. Introduction

As the information age rapidly advances, the von Neumann bottleneck problem stemming from the separation of storage and computing units has become more acute, leading to significant delays and power consumption [1,2,3]. Neuromorphic computing based on non-volatile memory (NVM) has gained significant attention due to its high energy efficiency and parallel-computing capability [4,5,6]. Hafnium oxide (HfO_2_) offers good CMOS compatibility and exceptional scalability, making it highly advantageous for next-generation non-volatile memory (NVM) technologies [7,8,9], such as FeRAM [10,11], FeFET [12,13], and FTJ [14,15]. Moreover, antiferroelectric HfO_2_ has been implemented in FTJ memory, highlighting its potential in NVM applications [16]. In recent years, the three-dimensional (3D) integration of these ferroelectric memories by vertically stacking memory devices has been successfully demonstrated, showing great potential to achieve ultrahigh memory density by maximizing cell area efficiency [17,18,19].

The polarization texture and ferroelectric dynamics can be theoretically explored by many simulation frameworks such as density function theory and phase field simulation [20,21]. For planar ferroelectric films, the application of vertical electric field perpendicular to the plane of planar ferroelectric films induces the formation of polar textures, including vortices [22], skyrmions [23], bubbles [24], and merons [25]. These polar textures are intricately related to a delicate balance between the bulk free energy, gradient energy, electrostatic energy, and elastic energy within the ferroelectric materials, depending on the device size and external excitation [26,27,28]. In recent years, the emergence of topological polar textures has been observed experimentally in perovskite ferroelectric films. Controlling these robust polar topological textures via electric fields holds promise for ultrahigh-density storage of ultrafine topological entities [29,30,31], paving the way for widespread applications in neuromorphic computing and broadening the scope of research in high-density, non-volatile memory.

A cylindrical-shell ferroelectric film is one of the key device structures to implement 3D memory integration using VNAND-like architecture, where the outer electrode is called plane electrode and the inner electrode is called pillar electrode [32]. However, compared with a planar ferroelectric film under a vertical electric field [22,23,24,25,26,27,28], polarization textures in such a cylindrical shell geometry-confined film that is biased via a radial electric field have barely been explored [33]. On the other hand, compared with that in perovskite ferroelectrics, topological polarization textures in HfO_2_ ferroelectrics are more or less uncovered. Recently, it has been revealed that cylindrical ferroelectric capacitors (FeCAPs) show distinct switching behaviors from planar devices [34]. Therefore, elucidating the polarization textures and switching dynamics in cylindrical HfO_2_ ferroelectrics is of great significance, to offer a deep understanding of ferroelectric memory devices such as FeRAM, FeFET, and FTJ, and also to provide theoretical guidance for emerging polar topological devices.

In this article, we investigate the polarization patterns and their switching dynamics within 3D nano cylindrical HfO_2_ ferroelectric devices, employing 3D phase field modeling. Section 2 describes the simulation method and calibration, where the 3D time-dependent Ginzburg–Landau (TDGL) and Poisson equations are self-consistently solved using the finite element method. Section 3 studies the topological polar domains and their switching dynamics as a function of the inner radius of the capacitor and the thickness of the ferroelectric layer. It is revealed that 3D cylindrical-shell ferroelectric capacitors demonstrate a size-controlled phase transition between ferroelectric (FE), antiferroelectric (AFE), and paraelectric (PE) phases. The emergence of these unique topological textures is analyzed via the intricate energy competition. Section 4 gives the conclusions.

## 2. Materials and Methods

**Figure 1:**
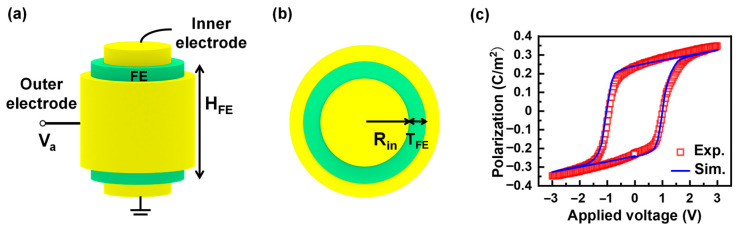
(**a**) Schematic of the 3D cylindrical MFM memory device. (**b**) Cross-section of x-y plane of MFM device. (**c**) Calibration of the simulated P-V characteristic of the HfO_2_-based FE MFM capacitor with the measured data.

Figure 1a,b illustrate the metal–ferroelectric–metal (MFM) film capacitor device with a 3D nano cylindrical-shell structure, with a ground bias applied to the inner electrode and an external time-dependent bias voltage applied to the outer electrode. The inner radius is denoted as *R_in_*; the thickness and height of the ferroelectric layer are *T_FE_* and *H_FE_*, respectively.

To study the spatial and temporal evolutions of polarization in ferroelectric films, the 3D TDGL and Poisson equations are self-consistently calculated within the ferroelectric region [35,36,37,38,39,40]. The total energy *F* in TDGL comprises bulk free energy *f_bulk_*, gradient energy *f_grad_*, elastic energy *f_elas_*, and electrostatic energy *f_elec_*,

(1) $$-\frac{1}{\Gamma }\frac{\partial P_{i}}{\partial t}=\frac{\partial F}{\partial P_{i}}\;(i=1,2,3)$$

(2) $$F=\int_{V}(f_{bulk}+f_{grad}+f_{elas}+f_{elec})dV$$

where the local polarizations are denoted as *P_i_* (*i* = 1, 2, 3), along with the *x* axis (*i* = 1), *y* axis (*i* = 2), and *z* axis (*i* = 3) in Cartesian coordinates. *t* represents the time, and Γ is a dynamic coefficient that describes the polarization reversal speed relative to the applied voltage [38].

The bulk free energy of the ferroelectric material is described by the LGD model [38],

(3) $$\begin{array}{cc} f_{bulk} & =\alpha_{1}(P_{1}^{2}+P_{2}^{2}+P_{3}^{2})\\ & +\alpha_{11}(P_{1}^{4}+P_{2}^{4}+P_{3}^{4})\\ & +\alpha_{12}(P_{1}^{2}P_{2}^{2}+P_{2}^{2}P_{3}^{2}+P_{1}^{2}P_{3}^{2})\\ & +\alpha_{111}(P_{1}^{6}+P_{2}^{6}+P_{3}^{6})+\alpha_{112}[P_{1}^{4}(P_{2}^{2}+P_{3}^{2})\\ & +P_{2}^{4}(P_{1}^{2}+P_{3}^{2})+P_{3}^{4}(P_{1}^{2}+P_{2}^{2})]+\alpha_{123}(P_{1}^{2}P_{2}^{2}P_{3}^{2}) \end{array}$$

where α_1_, α_11_, α_12_, α_111_, α_112_, and α_123_ are Landau coefficients used to describe the types of stable ferroelectric phases. Note that α_1_ is positive above the Curie temperature and negative below it.

The gradient energy, represented by the polarization gradient, characterizes the energy of dipole–dipole interaction arising from spatially non-uniform polarization [38],

(4) $$\begin{array}{cc} f_{G} & =\frac{1}{2}G_{11}(P_{1,1}^{2}+P_{2,2}^{2}+P_{3,3}^{2})+G_{12}(P_{1,1}P_{2,2}+P_{2,2}P_{3,3}+P_{1,1}P_{3,3})\\ & +\frac{1}{2}G_{44}[{\left(P_{1,2}+P_{2.1}\right)}^{2}+{\left(P_{2,3}+P_{3,2}\right)}^{2}+{\left(P_{1.3}+P_{3,1}\right)}^{2}]\\ & +\frac{1}{2}{G^{\prime }}_{44}[{\left(P_{1,2}-P_{2.1}\right)}^{2}+{\left(P_{2,3}-P_{3,2}\right)}^{2}+{\left(P_{1.3}-P_{3,1}\right)}^{2}] \end{array},$$

where *G*_11_, *G*_12_, *G*_44_, and $G_{44}^{\prime }$ are the gradient energy coefficients and $P_{i,j}=\partial P_{i}/\partial x_{j}$ [40].

The elastic strain energy density can be expressed as [38]

(5) $$\begin{array}{l} f_{elastic}=\frac{1}{2}C_{ijkl}(\varepsilon_{ij}-\varepsilon_{ij}^{0})(\varepsilon_{kl}-\varepsilon_{kl}^{0}) \end{array},$$

where *C_ijkl_* are the elastic stiffness tensor, *ε_ij_* are the total strain, and $\varepsilon_{ij}^{0}$ are the eigenstrain. The strain solution satisfies the boundary conditions and mechanical equilibrium as follows [38],

(6) $$\frac{\partial \sigma_{ij}}{\partial x_{j}}=0,$$

The elastic energy is given by the multiplication of the elastic stress [38],

(7) $$\sigma_{ij}=C_{ijkl}(\varepsilon_{ij}-\varepsilon_{ij}^{0}),$$

where *C*_11_, *C*_12_, and *C*_44_ are the independent elastic constants. The stress-free strain induced by the polarization field is described by the equation below [38],

(8) $$\begin{array}{c} \varepsilon_{11}^{0}=Q_{11}P_{1}^{2}+Q_{12}(P_{2}^{2}+P_{3}^{2}),\varepsilon_{23}^{0}=Q_{44}P_{2}P_{3}\\ \varepsilon_{22}^{0}=Q_{11}P_{2}^{2}+Q_{12}(P_{3}^{2}+P_{1}^{2}),\varepsilon_{13}^{0}=Q_{44}P_{1}P_{3}\\ \varepsilon_{22}^{0}=Q_{11}P_{3}^{2}+Q_{12}(P_{1}^{2}+P_{2}^{2}),\varepsilon_{12}^{0}=Q_{44}P_{1}P_{2} \end{array},$$

where *Q_ij_* are the electrostrictive coefficients.

Electrostatic energy, also called depolarization energy, can be expressed as [39,40,41]

(9) $$f_{elec}=-E_{i}P_{i}(i=1,2,3),$$

where the electric field *E_i_* within a ferroelectric is solved by the Poisson equation as follows [39,40,41]:

(10) $$\varepsilon_{0}\varepsilon_{FE}\nabla^{2}\varphi =\nabla \cdot P$$

where *ε*_0_ and *ε_FE_* are the vacuum and the relative permittivity, respectively, and *φ* is the electric potential. The inner and outer boundaries of the ferroelectric film are set as Dirichlet boundary conditions with *φ_in_* = 0 V and *φ_out_* = *V_a_*, where *V_a_* is the external applied voltage, while the top and bottom boundaries are set as Neumann boundary conditions.

The 3D TDGL and Poisson equations are self-consistently solved using the finite element method. The calibration of the *P*-*V* characteristic of the HfO_2_-based capacitor is shown in Figure 1c, where the simulated results are consistent with the measured data [42]. The good agreement between simulation and experiment verifies the coercive field and remnant polarization, with *E_c_* ~ 1 MV/cm and *P_r_* ~ 25 μC/cm^2^, which are typical values for HfO_2_ ferroelectrics, ensuring an accurate prediction of polar texture in HfO_2_ ferroelectrics. In the simulation, the mesh grid resolution is set as 1–2 nm for the three-dimensional cylindrical-shell structure, depending on the inner radius and ferroelectric thickness. The *V_a_* is set as a time-dependent ramp bias with triangular waveform, where the applied voltage amplitude and frequency are 3 V and 10 kHz, which are consistent with the measurement setup in [42]. The time step is set as *T*/100 with *T* = 1/*f*, where *T* and *f* are the period and frequency of the triangular waveform. The total simulation time is selected as 2*T*, and the simulation results in this work are derived from the second period. To start the self-consistent calculation of the TDGL and Poisson equations, the initial polarizations are set to zero in the whole ferroelectric region. In addition, the convergence criterion to end the self-consistent calculation is that the potential update satisfies |*φ^n^*^+1^ − *φ^n^*| < ε, where the convergence criterion ε is ~10^−4^. The structure parameter *H_FE_* is set as 10 nm, and *T_FE_* and *R_in_* are varied. The phase field parameters, which are listed in Table 1, are derived following the method in [42]. The temperature is set as 273 K in the simulation.

## 3. Results and Discussion

### 3.1. Phase Transitions Along with Topological Domain Patterns

**Figure 2:**
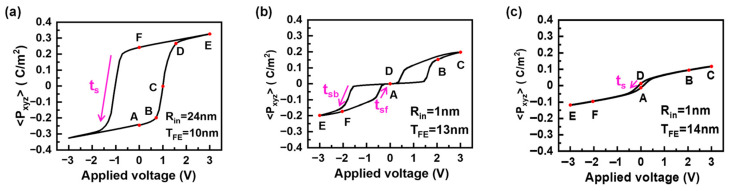
Polarization characteristics of cylindrical-shell HfO_2_ ferroelectric devices. (**a**) FE phase with *T_FE_* = 10  nm and *R_in_* = 24 nm, where the *P*-*V* curve shows a single hysteresis loop. (**b**) AFE phase with *T_FE_* = 13 nm and *R_in_* = 1 nm, where the *P*-*V* curve shows a double hysteresis loop and zero remnant polarization. (**c**) PE phase with *T_FE_* = 14 nm and *R_in_* = 1 nm, where the *P*-*V* curve shows a slim hysteresis and nearly linear characteristics. The corresponding switching time is labeled as *t_s_*. The points A–F during the polarization switching process will be discussed.

Figure 2 shows the polarization characteristics of cylindrical-shell HfO_2_ ferroelectric devices. It is observed that there a phase transition between FE, AFE, and PE phases with varied ferroelectric thickness and cylinder radius. A similar thickness-controlled phase transition in a planar ferroelectric thin film biased under vertical electric field is discussed in [40]. From Figure 2a, with *T_FE_* = 10  nm and *R_in_* = 24 nm, the *P*-*V* curve of the FE phase shows a single hysteresis loop and two stable remnant polarization states, which are two typical features of ferroelectrics. From Figure 2b, with *T_FE_* = 13 nm and *R_in_* = 1 nm, the *P*-*V* curve of the AFE phase shows a double hysteresis loop and zero remnant polarization. From Figure 2c, with *T_FE_* = 14 nm and *R_in_* = 1 nm, the *P*-*V* curve of the PE phase shows a slim hysteresis and nearly linear characteristics. Note that there is a very small and non-zero net remnant polarization in PE phase, which are consistent with the *P*-*V* curve of the experimental PE-phased HfO_2_ ferroelectrics [43,44,45].

**Figure 3:**
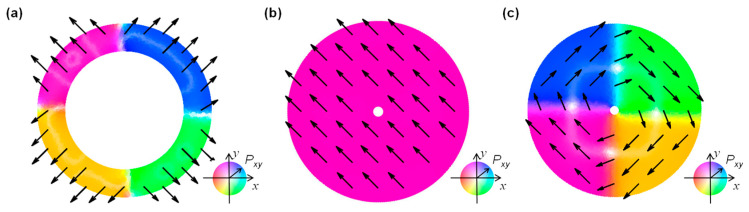
Topological domain patterns of in-plane polarization *P_xy_* in cylindrical-shell HfO_2_ ferroelectric devices at equilibrium state of *V_a_* = 0 V. (**a**) FE phase: Néel-like texture with center-type four-quad domain pattern. (**b**) AFE phase: monodomain pattern. (**c**) PE phase: Bloch-like texture with vortex four-quad domain. The arrows indicate the direction of polarization ***P**_xy_*.

To reveal the microscopic domain pattern under an in-plane radial electric field ***E*** *= (E_x_*, *E_y_*, 0*)*, namely, ***E*** = ***E**_xy_*, Figure 3 shows the topological domain patterns of in-plane polarization ***P**_xy_* in cylindrical-shell HfO_2_ ferroelectric devices at the equilibrium state of *V_a_* = 0 V, as the in-plane ***P**_xy_* is the dominant component of total polarization. From Figure 3a, ***P**_xy_* in the FE phase appears as a Néel-like texture. In particular, a center-type four-quad domain forms, and a quadruple domain wall in the FE case. From Figure 3b, ***P**_xy_* in AFE phases exhibit a monodomain-like pattern. The overall ***P**_xy_* gives rise to zero net polarization at equilibrium, as this circular-shape monodomain can be regarded as a double semicircle that has opposite polarization direction with respect to the center, one of which points towards the center <*P_xy_*_,_*_in_*>, and the other outwards from the center <*P_xy_*_,_*_out_*>. Therefore, zero net polarization in the AFE phase results from <*P_xy_*_,_*_in_*> = −<*P_xy_*_,_*_out_*>. This is analogous to the anti-parallel strip domains in planar AFE devices as discussed in [39,40], i.e., <*P_z_*_,_*_up_*> = −<*P_z_*_,_*_down_*>, thereby giving rise to zero net polarization. From Figure 3c, ***P**_xy_* in the PE phase exhibits another topological texture, i.e., a Bloch-like texture with the vortex four-quad domain pattern. The overall net ***P**_xy_* is very small compared with the FE phase, because ***P**_xy_* significantly deviates from the radial electric field direction.

### 3.2. Switching Dynamics of Ferroelectric Phase

To further understand the formation of center-type four-quad Néel-like domain in FE-phase cylindrical ferroelectrics, as shown in Figure 3a, Figure 3 describes the switching dynamics of in-plane polarization components including ***P**_x_*, ***P**_y_*, and ***P**_xy_*. When switched from negative to positive remnant polarization <*P_R_*>, the evolution of polarization distribution driven by in-plane ***E**_xy_* is clearly illustrated.

**Figure 4:**
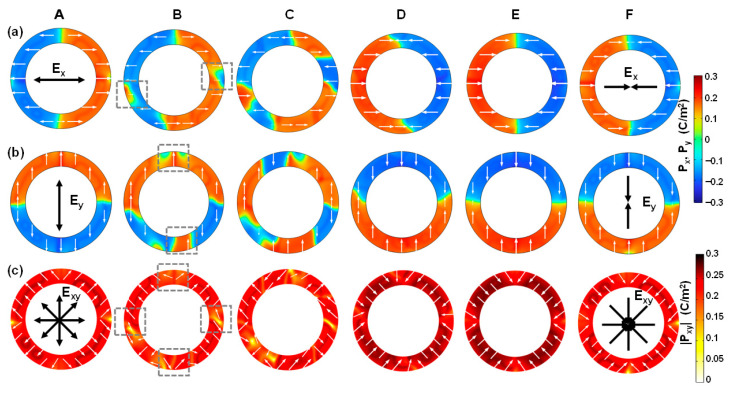
Polarization switching dynamics of the FE phase corresponding to Figure 2a, from negative to positive remnant polarization. Local distributions of (**a**) ***P**_x_*, (**b**) ***P**_y_*, and (**c**) ***P**_xy_* components, in which the FE phase is switched back and forth from the divergent to the convergent state. The in-plane *E_xy_* and its separate *E_x_* and *E_y_* components are illustrated at points A and F, clearly showing the electric field switching due to positive and negative triangular waveforms biased on the inner and outer electrodes of the cylindrical-shell structure (see Figure 1a). Note that the nucleation sites occurring at point B are marked with a dashed box.

Figure 4a shows the local ***P**_x_* distribution, namely, ***P**_x_*(*x*,*y*), where positive *P_x_* means that it points right and negative that it points left. In the negative <*P_R_*> case at equilibrium (point A), a double domain pattern within left and right semicircles forms, each of which direction points towards the outside of the cylindrical ferroelectric device. At the same time, a double 180° tail-to-tail DW*x* forms at the *x* = 0 interfaces. The domain wall width *W_DW_* is about 1.78 nm. When *V_a_* increases (point B), the reversed domains with inward direction nucleate at the outer edges at *y* = 0, which is marked with a dashed box. Based on the phase field simulation, the activation energy of ferroelectric reversal is calculated to be 0.3 eV. This value is within the reasonable range for HfO_2_ ferroelectrics [46]. Then, the reversed domains rapidly grow, going through the conditions when the total inward polarization <*P_x_*_,_*_in_*> is equal to the total outward polarization <*P_x_*_,_*_out_*>, namely, <*P_x_*_,_*_in_*> = <*P_x_*_,_*_out_*>, reaching zero net polarization at coercive voltage (point C). After this, the total polarization becomes positive, as the total inward polarization exceeds the total outward polarization. The growth of reversed domains stops until ***P**_x_* is fully switched inwards (point D). From point D to point E, the polarization intensity is strengthened by the response of background dielectrics. When we reach the positive <*P_R_*> case at equilibrium (point F), a double domain pattern with ***P_x_*** pointing inwards is stabilized, along with a double 180° head-to-head DW*_x_*.

Similarly, Figure 4b shows the local ***P**_y_* distribution, namely, ***P**_y_*(*x*,*y*), where positive *P_y_* means that it points upwards and negative that it points downwards. A double domain pattern within the top and bottom semicircles is stabilized at equilibriums, and a double 180° DW*_y_* forms at the *y* = 0 interfaces. There is an outward-directed polarization and tail-to-tail DW*_y_* in the negative <*P_R_*> case and an inward-directed polarization and head-to-head DW*_y_* in the positive <*P_R_*> case, in analogy to that of ***P**_x_* and DW*_x_*. The polarization reversal of ***P**_y_* proceeds similarly to the reversed domain nucleation and growth processes.

Figure 4c shows the local ***P**_xy_* distribution, namely, ***P**_xy_*(*x*,*y*) = (***P**_x_*(*x*,*y*), ***P**_y_*(*x*,*y*)). As a combination of ***P**_x_* and ***P**_y_*, ***P**_xy_* exhibits a center-type four-quad domain pattern. Meanwhile, a quadruple Néel-type 90° DW*_xy_* forms, due to a combined effect of a double 180° DW*_x_* and a double 180° DW*_y_*. The polarizations are inhomogeneous around the DW regions. This implies that the enhancement of gradient energy can alleviate the enhancement of electrostatic energy that is due to the strong depolarization field, thereby effectively suppressing the enhancement of total energy. This is the reason why the center-type four-quad domain pattern can be stabilized in cylindrical-shell ferroelectrics. Moreover, in-plane polarization ***P**_xy_* can be switched back and forth between convergent and divergent states driven by in-plane ***E**_xy_*, driven by the reversed domain nucleation and growth processes. To be specific, under *V_a_* with a frequency of 10 kHz, the switching time *t_s_* (see Figure 2a) is estimated to be 7 μs, and the corresponding domain wall velocity during polarization switching is about 0.067 m/s for the FE phase. Furthermore, the four-quad domain pattern of *P_xy_* switched between convergent and divergent states is consistent with the experimental observation of the planar cylindrical film biased under a vertical electric field by vector piezoresponse force microscopy (PFM) in [27]. In addition, the projection of two-semicircle *P_x_* and *P_y_*, obtained by our phase field simulation, is also consistent with the PFM measurements in [27]. This good agreement between experiments and simulation ensures that our results are reasonable.

### 3.3. Switching Dynamics of Antiferroelectric Phase

**Figure 5:**
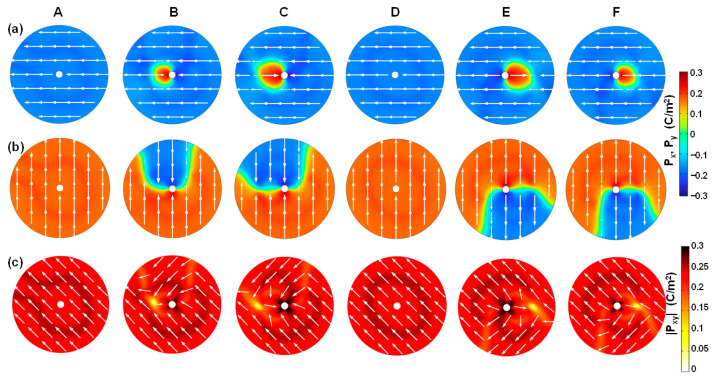
Polarization switching dynamics of the AFE phase corresponding to Figure 2b, from zero to negative or positive polarization. Local distributions of (**a**) ***P**_x_*, (**b**) ***P**_y_*, and (**c**) ***P**_xy_* components, when the AFE phase is switched back and forth from the monodomain to the dual-center domain pattern. The white arrows represent the polarization direction.

To further explore the emergent domain patterns in the AFE phase as shown in Figure 3b, Figure 5 describes the switching dynamics of in-plane polarization components including ***P**_x_*, ***P**_y_*, and ***P**_xy_* as labeled in Figure 2b.

Figure 5a shows the local ***P**_x_* distribution in the AFE phase. At equilibrium (point A), ***P**_x_* is uniformly distributed with identical directions parallel to the *x*-axis, implying that net ***P**_x_* is zero because overall <*P_x,in_*> = −<*P_x,out_*>, where *P_x_* in the left semicircle pointing right means inward *P_x_* (namely, *P_x_*_,_*_in_*), and *P_x_* in the right semicircle pointing right means outward *P_x_* (namely, *P_x_*_,_*_out_*). When positive voltage is applied (point B), ***P**_x_* begins to flip from the center on the left semicircle. The flipped area gradually expands as bias voltage increases (point C), leading to a reduction in *P_x_*_,_*_out_* and an increase in *P_x_*_,_*_in_*, and thereby an increase in net <*P_x_*_,_*_in_*>. Also note that the ***P**_x_* intensity close to the center is enhanced. Therefore, a dual-center domain forms with respect to ***P**_x_*, namely, a *P_x_*_,_*_in_* center and *P_x_*_,_*_out_* center, respectively. As the voltage decreases (point D), the flipped region progressively returns to its initial state and ultimately recovers to be identical to the original configuration at 0 V. A mirror-symmetry switching process takes place when negative voltage is applied (D-E-F-A).

Figure 5b shows the local ***P**_y_* distribution in the AFE phase. The ***P**_y_* distribution and switching dynamics are very similar to those of ***P**_x_*. At equilibriums, ***P**_y_* is uniformly distributed with identical directions parallel to the *y*-axis in response to ***E**_y_*. This results in zero net ***P**_y_* because overall <*P_y,in_*> = −<*P_y,out_*>, where *P_y_* in the upper semicircle pointing downwards means inward *P_y_* (namely, *P_y_*_,_*_in_*), and *P_y_* in the lower semicircle pointing downwards means outward *P_y_* (namely, *P_y_*_,_*_out_*). When positive or negative voltage is applied, ***P**_y_* in one of the upper and lower semicircles begins to flip from the center and gradually expands as bias voltage increases, while the ***P**_y_* intensity close to the center of the two semicircles is enhanced. Therefore, a dual-center domain forms with respect to ***P**_y_*, namely, a *P_y_*_,_*_in_* center and *P_y_*_,_*_out_* center, respectively. In addition, the domain wall width *W_DW_* is about 1.14 nm.

Figure 5c shows the local ***P**_xy_* distribution in the AFE phase, resulting from a combination of ***P**_x_* and ***P**_y_*. At equilibriums, in-plane ***P**_xy_* is almost uniform in terms of both intensity and direction. This is regarded as <*P_xy_*_,_*_in_*> = −<*P_xy_*_,_*_out_*>, where *P_xy_*_,_*_in_* is contributed by the upper-left semicircle and *P_xy_*_,_*_out_* by the lower-right semicircle. As the external voltage is biased, a dual-center emerges, where the ***P**_xy_* intensity of one of the two centers is enhanced, and the other one is weakened. At the same time, the weakened one radially moves away from the center to the edge, accompanied by an increase in the flipped area as the bias voltage increases. The domain pattern is switched back and forth between monodomain and dual-center domain depending on the equilibrium or non-equilibrium bias conditions. In particular, when *V_a_* varies with a frequency of 10 kHz, the switching time *t_s_* (see Figure 2b) is estimated to be 2 μs for forward switching (*t_sf_*) and 5 μs for backward switching (*t_sf_*)) in one of the double hysteresis loops in the AFE phase. The corresponding domain wall velocity during polarization switching is about 0.825 m/s for the AFE phase, which is about one order of magnitude higher than that of the FE phase.

### 3.4. Switching Dynamics of Paraelectric Phase

**Figure 6:**
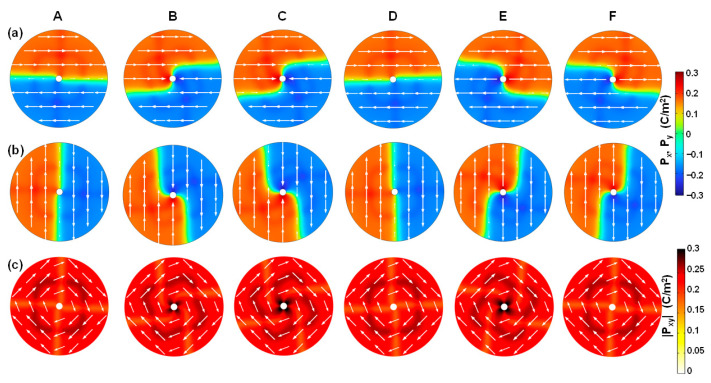
Polarization switching dynamics of the PE phase corresponding to (**a**), under varied applied voltage from zero to positive or negative polarization. Local distributions of (**a**) ***P***_x_, (**b**) ***P***_y_, and (**c**) ***P***_xy_ components, holding the Bloch-like four-quad domain pattern during switching.

To deeply understand the Bloch-like patterns in the PE phase as shown in Figure 3c, Figure 6 describes the switching dynamics of in-plane polarization components including ***P**_x_*, ***P**_y_*, and ***P**_xy_*, under varied applied voltage as labeled in Figure 2c.

Figure 6a shows the local ***P**_x_* distribution in the PE phase. At an initial 0 V (point A), ***P**_x_* in the upper semicircle is polarized right, while ***P**_x_* in the lower semicircle is polarized left. The overall circle is formed by two anti-parallel domains, thereby leading to nearly zero remnant polarization. At the same time, there is an Ising-type DW*_x_* parallel to the *x*-axis. The domain wall width *W_DW_* is about 0.84 nm in the PE phase. Note that the two DWs located left and right are not fully aligned, and the slim misaligned regions around the DW*_x_* give rise to a very small amount of net <*P_x_*_,_*_out_*>. When a positive voltage is applied (points B and C), the two Ising-DW start to move anticlockwise. Consequently, the net polarization changes from net <*P_x_*_,_*_ou_*_t_> to net <*P_x_*_,_*_in_*>. When the voltage returns to 0 V (point D), the DW*_x_* move clockwise, and polarization recovers to a state similar to the initial one with a small amount of net <*P_x_*_,_*_in_*>. A mirror-symmetry switching process takes place when negative voltage is applied.

Figure 6b shows the local ***P**_y_* distribution in the PE phase. The two vertical DW*_y_* move anticlockwise or clockwise, depending on the voltage polarity. The polarization is switched between a small amount of net <*P_y_*_,_*_out_*> and net <*P_y_*_,_*_in_*>. As the ***P**_y_* distribution and switching dynamics are very similar to those of ***P**_x_* with a rotation of 90°, they are not described to avoid repetition.

Figure 6c shows the local ***P**_xy_* distribution in the PE phase. At 0 V, the remnant polarization is weak, because the vortex-like four-quad domains are polarized far from the radial direction, and are nearly tangent to the radial direction. This means that the polarization direction is tangent to the electric field direction. As bias voltage is increasingly applied, the vortex-like domain moves as a whole and rotates slightly off the tangent direction. Similarly to that of FE and AFE phases, the switching time *t_s_* (see Figure 2c) and the domain wall velocity in the PE phase are derived to be 6 μs and 0.2 m/s, respectively.

### 3.5. Energy Competition in Different Phases

**Figure 7:**
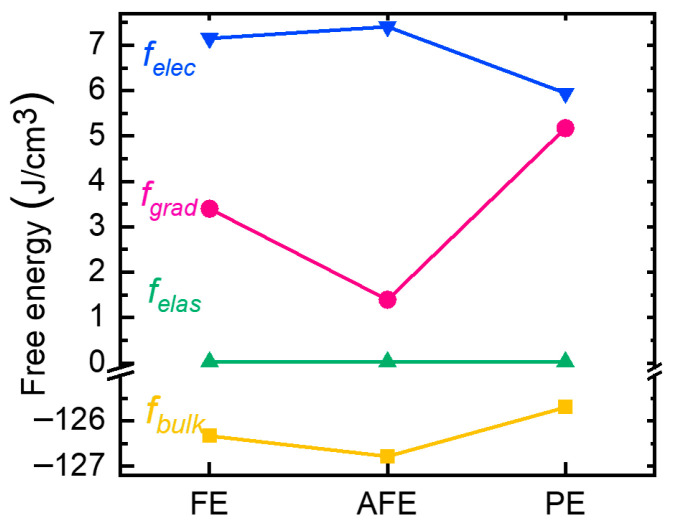
Comparison of various free energy components in FE, AFE, and PE phases at equilibriums.

The polarization characteristics of the FE, AFE, and PE phase transitions highlight the complex dynamic behaviors in cylindrical-shell ferroelectrics under external radial electric fields. These distinct polarization patterns, including Néel-like, monodomain, and Bloch-like configurations, are deeply rooted in the intrinsic energy competition. Hence, Figure 7 gives the energy variation in the abovementioned FE, AFE, and PE phases, including *f_bulk_*, *f_grad_*, *f_elec_*, and *f_elas_*, at equilibriums, the corresponding domain textures of which are shown in Figure 3.

**Figure 8:**
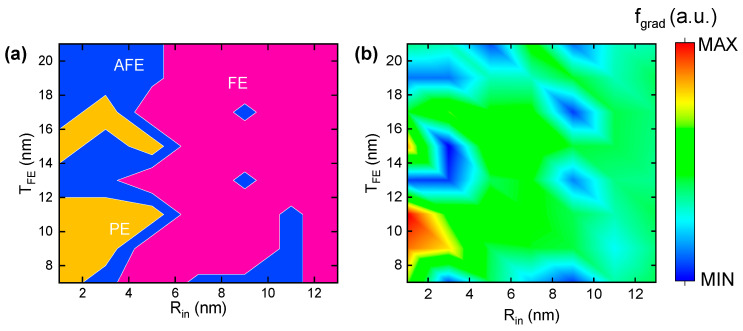
Size-controlled *R_in_*-*T_FE_* (**a**) phase and (**b**) *f_grad_* diagram for cylindrical ferroelectrics.

Furthermore, Figure 8a gives the size-controlled *R_in_*-*T_FE_* phase diagram for the cylindrical MFM ferroelectric capacitor to fully capture the phase transition between FE, AFE, and PE. From Figure 7, *f_grad_* between the three phases changes most. Therefore, the related energy diagram of *f_grad_* as a function of *R_in_*-*T_FE_* is presented in Figure 8b. The relative value of *f_bulk_* between FE, AFE, and PE in Figure 7 is consistent with [47], where the AFE phase has relatively low *f_bulk_*, followed by the FE and PE phases. The energy competition between FE, AFE, and PE based on Figure 7 and Figure 8 is discussed in the following paragraphs.

In the FE phase, where *R_in_* is comparably larger than *T_FE_*, the cylindrical ferroelectric capacitor behaves like the planar thin film one, and the formation of a domain wall can effectively relieve the strong depolarization effect. Therefore, the gradient energy increases, but the electrostatic energy decreases. Also noteworthy are the vertical 180° strip domains that exist in planar thin film ferroelectrics; their domain size changes as ferroelectric thickness varies [39,40]. In contrast, the center-type four-quad domain pattern of the topological polar texture is stabilized in cylindrical structures, which are topologically protected due to geometry confinement, meaning that they are independent of *R_in_* and *T_FE_* as long as they are in the FE phase. Such topological polar textures are energetically favorable as the bulk free energy remains much lower in the relatively deep double wall with the appearance of remnant polarization.

In the AFE phase, where *R_in_* is smaller than *T_FE_*, the curvature asymmetry between inner and out boundaries is greatly enhanced. The evolution of the domain wall vanishes towards equilibrium; otherwise, the gradient energy would be much higher and the domain pattern would become unstable. The formation of a quasi-monodomain without DW is actually a bidomain that has anti-parallel opposite directions inwards and outwards from the center, giving rise to a zero net polarization. The bidomain pattern is attributed to the increased electrostatic energy that is determined by anti-parallel inhomogeneous polarization [48]. In other words, the decrease in gradient energy is dominant compared with the increase in electrostatic energy, making the AFE phase stable.

In the PE phase, where *T_FE_* is large and *R_in_* is small, polarization with tangent directions suggests that the gradient energy is significantly high in such ultra-nonuniform conditions. This explains the formation of a vortex-like four-quad domain in the PE phase, with a very thick domain wall compared with the FE phase. Additionally, the great thickness of the ferroelectric layer reduces the overall electric field, making the electrostatic energy remarkedly lower.

## 4. Conclusions

We have systematically investigated the phase transitions, polarization topologies, and switching dynamics within three-dimensional cylindrical nano-ferroelectric devices using a time-dependent phase field model. The interplay of bulk free energy, gradient energy, depolarization energy, and elastic energy is critical in determining the phase behavior and stability of these devices. We have demonstrated that by controlling key size parameters such as inner radius and ferroelectric layer thickness, it is possible to induce phase transitions between ferroelectric, antiferroelectric, and paraelectric phases, each of which exhibits distinct topological patterns including Néel-like, monodomain, and Bloch-like textures. These findings underscore the potential of cylindrical ferroelectric devices to be used in high-density non-volatile memory and neural computing devices, and the ability to manipulate topological domains via geometry engineering offers exciting possibilities for device design and functionality. Future research should focus on further observation of these topological polar textures by advanced measurement techniques and of the origins of these FE, AFE, and PE phases by density function theory, as well as optimizing these devices for practical applications, exploring their integration into larger memory architectures, and investigating the dynamic control of their topological states for enhanced performance in real-world environments.

## Figures and Tables

**Table 1:** Parameters used in the phase field simulation.

Parameters	Values	Units
α_1_	−2.5 × 10^9^	V·m/C
α_11_	−2 × 10^8^	V·m^5^/C^3^
α_12_	5 × 10^8^	V·m^5^/C^3^
α_111_	3.2 × 10^11^	V·m^9^/C^5^
α_112_	3.5 × 10^11^	V·m^9^/C^5^
α_123_	3.5 × 10^11^	V·m^9^/C^5^
G_11_	1 × 10^−9^	V·m^3^/C
G_12_	0	V·m^3^/C
G_44_	1 × 10^−9^	V·m^3^/C
G′_44_	1 × 10^−9^	V·m^3^/C
C_11_	450.129	GPa
C_12_	124.003	GPa
C_44_	5.469	GPa
*Q*_11_	0.0056	m^4^/C^2^
*Q*_12_	−0.0059	m^4^/C^2^
*Q*_44_	0.0385	m^4^/C^2^
*Γ*	0.01	S/m
*ε*_FE_	30	1
*f*	10	kHz
*H_FE_*	10	nm

## Data Availability

The original contributions presented in this study are included in the article. Further inquiries can be directed to the corresponding author.
